# DECODE-3DViz: Efficient WebGL-Based High-Fidelity Visualization of Large-Scale Images using Level of Detail and Data Chunk Streaming

**DOI:** 10.1007/s10278-025-01430-9

**Published:** 2025-02-14

**Authors:** Mohammed A. AboArab, Vassiliki T. Potsika, Andrzej Skalski, Maciej Stanuch, George Gkois, Igor Koncar, David Matejevic, Alexis Theodorou, Sylvia Vagena, Fragiska Sigala, Dimitrios I. Fotiadis

**Affiliations:** 1 Unit of Medical Technology and Intelligent Information Systems, Dept. of Materials Science and Engineering, University of Ioannina, 45110 Ioannina, Greece; 2 Electronics and Electrical Communication Engineering Dept, Faculty of Engineering, Tanta University, Tanta, Egypt; 3 Dept. of Measurement and Electronics, AGH University of Krakow, 30-059 Krakow, Poland; 4 MedApp S.A, 30-037 Krakow, Poland; 5 Clinic for Vascular and Endovascular Surgery, University Clinical of Serbia, Belgrade, Serbia; 6 Faculty of Medicine, University of Belgrade, Belgrade, Serbia; 7 First Propaedeutic Dept. of Surgery, National and Kapodistrian University of Athens, Athens, Greece; 8 Biomedical Research Institute, Foundation for Research and Technology-Hellas, University Campus of Ioannina, 45110 Ioannina, Greece

**Keywords:** Diagnostic imaging, GPU optimization, Interactive 3D visualization, Peripheral artery CT imaging, Progressive chunk streaming, Volume rendering

## Abstract

The DECODE-3DViz pipeline represents a major advancement in the web-based visualization of large-scale medical imaging data, particularly for peripheral artery computed tomography images. This research addresses the critical challenges of rendering high-resolution volumetric datasets via WebGL technology. By integrating progressive chunk streaming and level of detail (LOD) algorithms, DECODE-3DViz optimizes the rendering process for real-time interaction and high-fidelity visualization. The system efficiently manages WebGL texture size constraints and browser memory limitations, ensuring smooth performance even with extensive datasets. A comparative evaluation against state-of-the-art visualization tools demonstrates DECODE-3DViz's superior performance, achieving up to a 98% reduction in rendering time compared with that of competitors and maintaining a high frame rate of up to 144 FPS. Furthermore, the system exhibits exceptional GPU memory efficiency, utilizing as little as 2.6 MB on desktops, which is significantly less than the over 100 MB required by other tools. User feedback, collected through a comprehensive questionnaire, revealed high satisfaction with the tool's performance, particularly in areas such as structure definition and diagnostic capability, with an average score of 4.3 out of 5. These enhancements enable detailed and accurate visualizations of the peripheral vasculature, improving diagnostic accuracy and supporting better clinical outcomes. The DECODE-3DViz tool is open source and can be accessed at https://github.com/mohammed-abo-arab/3D_WebGL_VolumeRendering.git.

**Supplementary Information:**

The online version contains supplementary material available at 10.1007/s10278-025-01430-9.

## Introduction

The field of medical imaging has undergone significant advancements, particularly in the visualization of complex volumetric data derived from imaging modalities such as computed tomography (CT) and magnetic resonance imaging (MRI). These technologies have become crucial in the diagnosis and treatment of various medical conditions, including peripheral artery disease (PAD) [1]. Traditionally, the visualization of medical data has been confined to specialized visualization stations available mainly in radiology departments [2, 3]. This limited access poses a significant barrier to other doctors, who need quick access to three-dimensional data visualization. Therefore, there is a growing need for web-based applications that allow broader and more flexible access to medical imaging data.

PAD is characterized by narrowing or blockage of arteries, primarily affecting the lower extremities. The small pathologies associated with PAD require high-resolution imaging and precise visualization to ensure accurate diagnosis and treatment planning. The size and complexity of PAD datasets make it challenging for currently available solutions to provide the necessary level of detail (LOD) and interactivity, especially in a web-based environment [4, 5].

WebGL, a JavaScript API for rendering interactive 3D graphics within web browsers, provides a promising solution to these challenges. By leveraging WebGL for 3D volume rendering, it is used to visualize complex volumetric data without the need for additional plugins, thus ensuring broader accessibility and usability [6]. This approach provides an accessible platform for medical professionals to interact with detailed 3D models of medical images in real time. Furthermore, recent studies have demonstrated that WebGL can effectively render 3D medical datasets, offering significant potential for improving medical diagnosis and treatment planning [7–9]. Despite these advantages, implementing 3D volume rendering for large-scale medical datasets in WebGL is beset with challenges. These include WebGL texture size constraints, browser memory allocation, and the impact of large datasets on browser performance. Addressing these challenges requires innovative solutions which optimize the visualization pipeline, ensuring high performance and visual fidelity​​.

## Related Work

Recent advancements in 3D WebGL volume rendering have been pivotal in enhancing medical data visualization, although they still face significant challenges. Zhang (2019) [10, 11] contributed notably to projects that enabled real-time visualization and interactive data management; however, these efforts were constrained by WebGL2's limited data storage capacity, especially for handling extensive medical datasets. In addition, Lajara et al*.* (2019) [12] sought to improve the efficiency of web-based visualization by implementing a pyramidal structure in the middleware layer to expedite frame transmission. Nonetheless, their approach still encountered scalability issues with larger datasets. For advanced rendering techniques, Visutsak et al*.* (2020) [13] utilized 3D surface rendering techniques, such as marching cubes and histogram pyramids, but their method suffers from issues such as surface roughness due to unused voxels. Furthermore, Xu et al*.* (2022) [14] introduced cinematic volume rendering (CVR) within browsers, which showed promise but was hampered by significant memory limitations. In addition, Li et al*.* (2023) [15] developed a framework for real-time medical image rendering and 3D visualization, incorporating advanced interpolation techniques to manage missing voxels; however, this system requires further improvements in memory management and rendering efficiency. For multiresolution and adaptive techniques, Zhu et al*.* (2023) [16] advanced the field with an adaptive resolution enhancement method using eye tracking, allowing dynamic resolution adjustments on the basis of the viewer's focus, thereby improving analytical efficiency. Moreover, Kumar et al*.* (2024) [8] presented RadVolViz, a tool designed to enhance visual differentiation through advanced color mapping techniques in WebGL; however, it needs optimization for handling large datasets effectively​​.

These studies collectively underscore the persistent challenges in managing large datasets, optimizing memory usage, and ensuring high-quality rendering. Our work seeks to address these limitations through the development of an open source DECODE-3DViz pipeline [17], which offers a robust and efficient solution for high-fidelity visualization of peripheral artery CT images.

## Research Objective

To address the challenges identified in the literature, our work seeks to develop innovative solutions that optimize the visualization pipeline through the application of an LOD algorithm. The specific objectives are as follows:

**RO1:** Efficiently manage WebGL texture size limitations by developing techniques that utilize the LOD algorithm to render large peripheral artery CT datasets without performance degradation or errors, thereby overcoming texture size constraints. **RO2:** Implement strategies to prevent memory allocation errors, employing the LOD algorithm to ensure the complete and accurate rendering of high-resolution medical imaging data. **RO3:** Develop a method for chunk streaming large datasets, preventing browser crashes and maintaining application responsiveness and usability. **RO4:** Establish an approach to downsample only when necessary, guided by the LOD algorithm, to preserve as much detail as possible in the rendered images and maintain high visual fidelity. **RO5:** Provides functionality for specifying and rendering regions of interest (ROIs) in their original resolution using the LOD algorithm, ensuring that critical volumes are visualized in high detail for accurate medical diagnosis.

The overarching aim of these objectives is to increase the performance, accuracy, and usability of web-based applications for visualizing large-scale peripheral artery CT imaging datasets. This will ultimately support improved diagnostic outcomes and advance the field of medical imaging technology.

## Approach and Implementation

### Data Acquisition

The dataset used to evaluate DECODE-3DViz includes CT scans from 22 patients diagnosed with PAD. These cases were randomly selected from the hospital imaging archive to ensure diverse representations of PAD severity levels, ranging from mild to advanced cases. The selection process prioritized anatomical variability and imaging consistency. However, as a hospital-based dataset, it primarily includes patients with moderate to severe PAD, potentially underrepresenting early-stage cases.

All the scans were acquired via a Revolution EVO CT scanner (GE Healthcare) in helical acquisition mode, ensuring high spatial resolution. The imaging protocol included a 512 × 512 resolution, 16-bit depth, and slice counts ranging from 255 to 2305 slices. The key acquisition parameters were a slice thickness of 0.625 mm, pitch of 0.984:1, tube voltage of 120 kVp, and an automatic exposure control system for optimized image quality while minimizing the radiation dose. This standardized imaging setup ensures consistency across the dataset, enabling a reliable performance evaluation of DECODE-3DViz.

The research protocol for using these CT scans was approved under ethical committee protocol numbers 9876/28.3.24 and 11,293/9.4.24. The study was conducted within the radiodiagnostic department at the General Hospital of Athens, ensuring adherence to ethical standards for clinical research.

### System Design

**Fig. 1:**
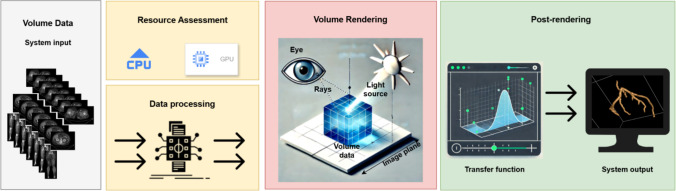
Workflow of 3D WebGL Volume Rendering for Peripheral Artery CT Imaging

The system design of DECODE-3DViz for 3D WebGL volume rendering of peripheral artery CT images involves a five-stage workflow: volume data input, resource assessment, data processing, volume rendering, and postrendering, as shown in Fig. 1. The system begins by assessing and configuring processing resources through resource assessment, where the central processing unit (CPU) is primarily responsible for task allocation, data handling, and managing heap memory to optimize performance. The graphics processing unit (GPU) is configured for high-performance rendering, with constraints such as MAX_3D_TEXTURE_SIZE carefully considered to ensure efficient handling of large textures. The workflow ensures that computational demands are met while maintaining efficient memory and resource utilization.

Initially, CT slices are collected to form a detailed volumetric dataset, which undergoes resource assessment to evaluate computational requirements and configure processing resources efficiently. During data processing, the dataset is prepared using techniques such as data chunking and an LOD algorithm, which dynamically adjusts the resolution to manage large datasets efficiently. The volume rendering stage uses WebGL to cast rays through the data, creating a 3D representation with shading and lighting for enhanced visual realism. Volume clipping and interaction techniques focus on specific ROIs to improve clarity and detail. In the final postrendering stage, a transfer function maps data values to colors and opacities, allowing interactive adjustments for detailed visualization and accurate diagnosis.

### Preprocessing Pipeline

**Fig. 2:**
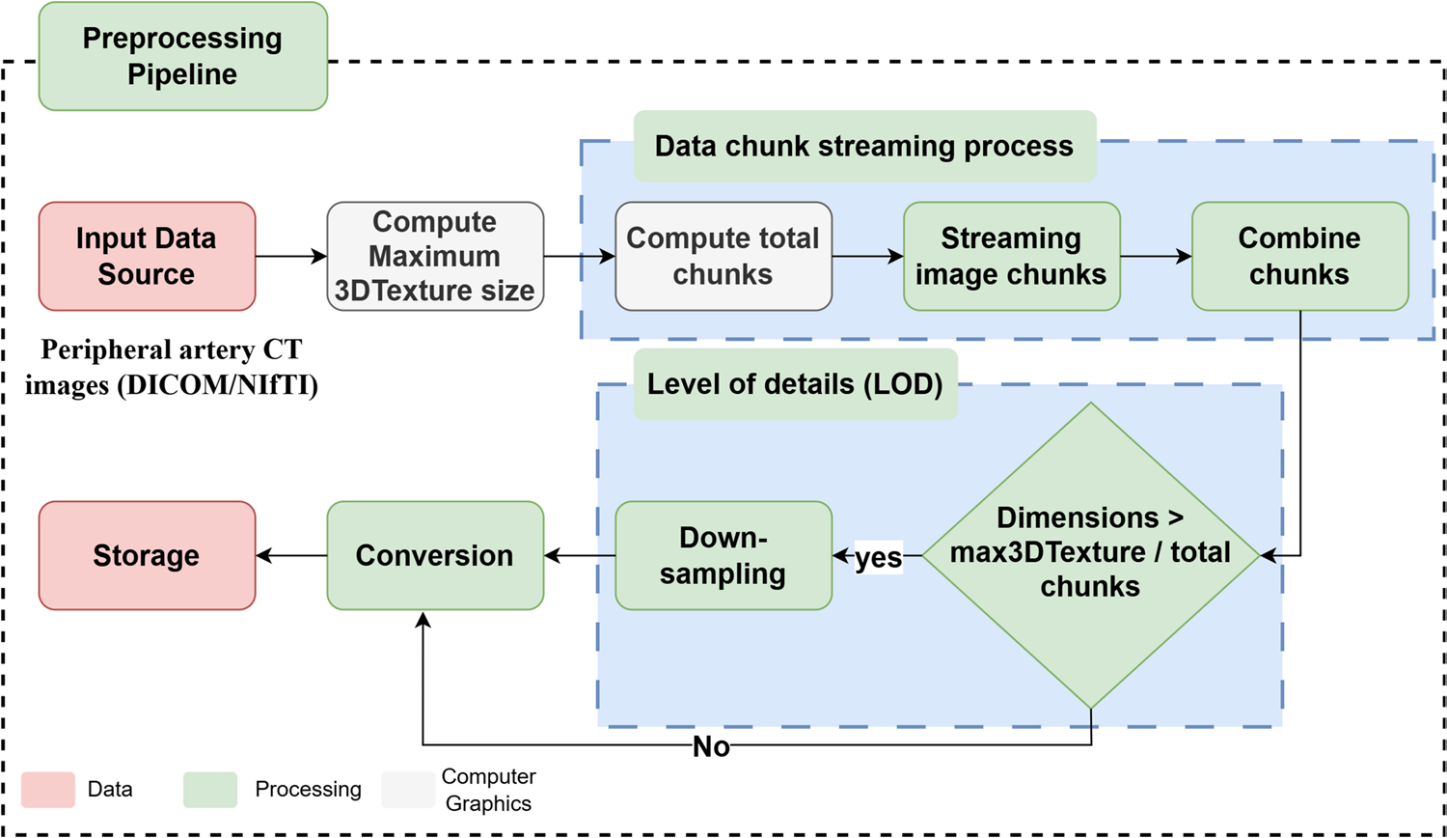
Schematic Diagram of the Preprocessing Pipeline for 3D WebGL Volume Rendering

The preprocessing pipeline of DECODE-3DViz consists of several essential steps for effective rendering. This section outlines the process starting from source data through the computation of the maximum 3D texture size, computation of total chunks required for rendering, streaming image chunks, combining chunks, and applying the LOD algorithm, which includes downsampling if necessary, as it is shown in Fig. 2.

1. Input Data Source

The source data comprises peripheral artery CT images in DICOM or NIfTI formats [18, 19]. These images are volumetric datasets that require processing to be rendered using WebGL technology. The importance of using both DICOM and NIfTI formats lies in their widespread adoption in medical imaging and their ability to store complex image data with metadata, which is crucial for accurate visualization and analysis. The data are loaded into the browser environment where WebGL is used for rendering.

2) Maximum 3D Texture Size

Determining the maximum 3D texture size supported by WebGL 2.0 is crucial for assessing the WebGL context's ability to handle 3D textures. This process involves querying the *MAX_3D_TEXTURE_SIZE* parameter, which specifies the largest dimension in pixels for each axis of a 3D texture. Understanding that limitation is vital for partitioning volumetric data into manageable chunks, ensuring efficient rendering and optimal performance [15, 20]. In addition, optimizing the upload process involves managing the available JavaScript heap memory using the *performance.memory* API, with 75% of the total heap size typically allocated for texture uploads. In cases where this API is not supported, a fallback value is used to maintain a balance between maximizing memory usage for uploads and ensuring sufficient memory for other operations [21–23].

3) Data Chunk Streaming Process

The data chunk streaming process is critical for the efficient handling and rendering of volumetric datasets in a WebGL environment. To manage large volumes of data, the dataset is initially divided into manageable chunks. This division is guided by constraints such as the maximum 3D texture size supported by the WebGL context and the available upload memory. A chunk factor, typically set as a fraction (e.g., 0.25) of the maximum 3D texture size, determines the chunk size, ensuring that the chunks remain within feasible limits. The chunk size is computed as follows:

1 $$chunkSize=\text{min}\left(\left\lfloor max3DTextureSize\times chunckFactor\right\rfloor\text{,}\left\lfloor\frac{maxUploadMemoryBytes}2\right\rfloor\right)$$

where *max3DTextureSize* represents the maximum allowable texture size along each dimension, *chunkFactor* is the fraction determining the size of each chunk, and *maxUploadMemoryBytes* is the available memory for uploading data. The total number of chunks required to process the dataset, denoted *totalChunks*, is determined by dividing the total depth of the dataset by the chunk size and rounding up:

2 $$totalChunks= \lceil\frac{depth}{chunkSize}\rceil$$

where *depth* is the depth of the dataset along the axis being partitioned. This calculation ensures that all the data slices account for [24–26].

In the data chunk streaming phase, the volumetric data are dynamically partitioned into these chunks for incremental processing, which optimizes memory usage and enables smooth visualization. For each chunk, the starting and ending positions along the depth axis are calculated, and data from the original dataset are extracted accordingly. Each voxel within a slice is mapped to the corresponding position in a new array, preserving spatial relationships. The assembled chunks, containing the extracted data with updated dimensions, are then added to a list of chunks to ensure complete coverage of the dataset [27–29].

The chunks are later combined into a single volumetric dataset, which is essential for reconstructing the original volume. This process assumes a consistent width and height across all chunks, as derived from the source data. The total depth of the combined dataset, denoted by *totalDepth*, is calculated by summing the individual depths of all chunks:

3 $$totalDepth= \sum_{i=1}^{n}{depth}_{i}$$

where $n$ is the total number of chunks, and ${depth}_{i}$​ is the depth of each individual chunk. This approach maintains the correct spatial relationships and ensures that the final dataset is ready for subsequent processing or rendering. If the combined dataset exceeds the maximum 3D texture size per chunk, an LOD algorithm is applied, as it is shown in Fig. 2, to keep the dataset within these constraints and optimize it for efficient rendering.

4) Level of detail (LOD)

LOD adjustment is essential for efficient rendering within the constraints of the WebGL context, particularly for managing large volumetric datasets. This adjustment involves reducing the dataset’s resolution while preserving critical features. The maximum dimension of the dataset is compared to a target maximum dimension to calculate the LOD adjustment factor. If the dataset dimensions exceed the target, an adjustment factor, *maxFactor*, is determined by the maximum ratio of the dataset dimensions to the target dimension:

4 $$maxFactor=\text{max}\left(\frac{dimensions}{targetMaxDimension}\right)$$

where the *dimensions* represent the actual dimensions of the dataset, and *targetMaxDimension* is set to half of the maximum 3D texture size:

5 $$targetMaxDimension= \frac{max3DTextureSize}{2}$$

The dataset is then resized by dividing each dimension by *maxFactor* and rounding up to the nearest integer, with the voxel spacing adjusted accordingly to maintain spatial relationships [30, 31]. When any dimension of the combined dataset surpasses the maximum 3D texture size divided by the total number of chunks, downsampling becomes necessary. This downsampling process reduces the dataset's resolution while retaining essential features and uses trilinear interpolation to maintain data integrity [32–34]. Each voxel in the downsampled dataset is calculated by interpolating values from the original dataset on the basis of the indices and weights of the surrounding voxels. The outcome is a dataset with optimized dimensions and voxel spacing suitable for efficient rendering in a WebGL environment, as it is illustrated in the LOD algorithm in Fig. 2.

5) Conversion and Storage

The process of data storage and conversion is crucial for preparing downsamples via LODs or original combined datasets for efficient rendering. This involves converting the dataset from formats such as DICOM or NIfTI to the VTK image data format [35–37]. This conversion is necessary for compatibility with WebGL volume rendering, specifically when vtk.js is utilized [38]. The converted data are stored as VTK images, which are optimized for efficient rendering in WebGL.

### WebGL Volume Rendering Pipeline

**Fig. 3:**
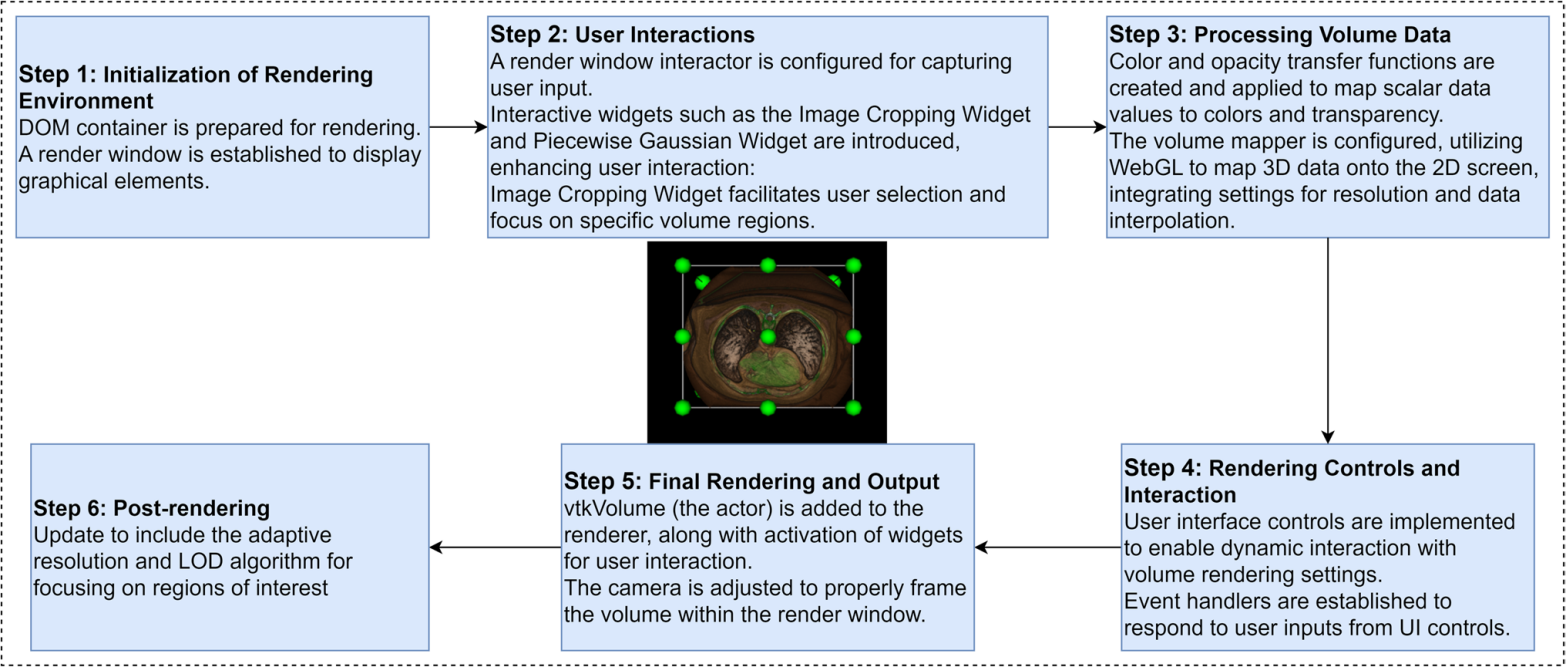
WebGL Volume Rendering Pipeline with Adaptive Resolution and LOD Algorithm

The WebGL volume rendering pipeline is meticulously structured into several stages, each playing a crucial role in generating high-quality, interactive 3D visualizations of peripheral artery CT images. This systematic approach, as it is illustrated in Fig. 3, ensures an organized workflow, enabling efficient and effective rendering.

1. Initialization of the rendering environment

After preprocessing and conversion, the dataset is prepared for rendering. This involves acquiring input image and color data and setting up the core visualization components, including a rendering window, a renderer, and an OpenGL rendering window. The rendering container is identified within the HTML document, establishing the essential environment for visualization [39, 40]. In addition, an interactor is initialized to facilitate responsive and interactive user experiences [41, 42].

2) User Interactions

User interaction is further enhanced through a graphical overlay that provides visual feedback and a widget manager that handles interactive widgets, such as image cropping tools, allowing dynamic modification of rendering parameters with real-time updates [14, 43]. Interactive widgets, including the image cropping width and piecewise Gaussian width, support user interaction by facilitating selection and focusing on specific volume regions.

3) Volume Render Processing

In the volume rendering processing phase, detailed volume rendering settings are configured to accurately represent the CT data. This involves setting up volume properties such as color and opacity transfer functions, shading parameters, and interpolation types. The volume mapper is linked to the input image data to ensure precise visualization [44, 45].

A critical component of this phase is the transfer function, which controls the visualization of CT data in terms of color and opacity. The main volume rendering is as follows [10, 46, 47]:

6 $$I\left(D\right)= {I}_{0}{e}^{-{\int }_{0}^{D}\tau \left(t\right)dt}+{\int }_{0}^{D}{e}^{-{\int }_{s}^{D}\tau \left(t\right)dt}\tau \left(s\right)C\left(s\right)ds,$$

where $I\left(D\right)$ is the intensity of the light after passing through the volume at depth $D$, ${I}_{0}$ is the initial intensity of the light, $\tau \left(t\right)$ is the optical depth or attenuation coefficient at position $t$, and $C\left(s\right)$ is the color or emission at position $s$. This equation accounts for both absorption and emission within the volume, which is critical for accurate volume rendering. The transfer function is defined as follows [11, 48]:

7 $$\begin{array}{c}T\left(\upsilon \right)=(\alpha \left(\upsilon \right), c\left(\upsilon \right))\\ \alpha \left(\upsilon \right)= {\sum }_{i=1}^{n}{w}_{i}G\left(\upsilon -{\upsilon }_{i},{\sigma }_{i}\right)\\ c\left(\upsilon \right)= {\sum }_{i=1}^{n}{c}_{i}L\left(\upsilon -{\upsilon }_{i}\right)\end{array}$$

where $\alpha \left(\upsilon \right)$ is the opacity transfer function and $c(\upsilon )$ is the color transfer function, $G$ is a Gaussian function centered at ${\upsilon }_{i}$ with width ${\sigma }_{i}$, ${w}_{i}$ represents the weights, $L$ is a linear function centered at ${\upsilon }_{i}$, and ${c}_{i}$ represents the color values associated with intensity ${\upsilon }_{i}$.

To facilitate interactive adjustments, a *vtkPiecewiseGaussianWidget* [38] is created and configured within a dynamically generated HTML container. This widget allows users to modify the color and opacity mappings interactively. A histogram is generated from the CT data values, providing a visual representation of the data distribution. This histogram is used to set up the data array for the widget, enabling users to see and adjust how data values are mapped to colors and opacities. The *vtkPiecewise function* [38] is used to define the opacity transfer mapping, setting specific points to make different tissue types transparent or opaque. Simultaneously, the *vtkColorTransferFunction* [38] assigns colors to different intensity values, enhancing the visual distinction between various tissues, such as air, lung, fat, muscle, and bone. The widget’s opacity change events are closely monitored, and any user adjustments are dynamically applied to the transfer functions. This allows for real-time updates to the rendered volume representation, ensuring that users can fine-tune the visualization parameters to achieve the best possible representation of the CT data.

4) Rendering Controls and Interaction

In this stage, interactive features are meticulously refined to enhance user engagement and control. Advanced manipulators for pan, zoom, and rotation operations are seamlessly integrated, providing users with comprehensive control over the viewing experience. An orientation marker widget is added to facilitate spatial orientation within the 3D scene, ensuring that users can navigate the volume data effectively [49, 50]. In addition, control panels are integrated to allow precise adjustments to various rendering parameters, including gradient opacity, scalar opacity, sample distance, blending modes, visibility, and shading of the volume. These controls enable users to customize the visualization to meet specific diagnostic needs, thereby enhancing the overall utility and effectiveness of the rendered images.

5) Final Rendering and Output

The final rendering stage involves applying the configured settings and controls to produce the visual output. The rendering pipeline integrates mechanisms for updating the rendering in response to changes in cropping planes, utilizing *vtkPlane* [38] instances for precise clipping operations. The sophisticated color and opacity transfer functions established during volume processing are crucial, ensuring that different tissue types are accurately represented and visually distinct. Initial rendering involves downsampling for performance optimization on the basis of the LOD algorithm. The final color ${C}_{final}$​ along a ray can be computed using the integral as:

8 $${C}_{final}= {\int }_{{t}_{0}}^{{t}_{1}}c\left(t\right) \alpha \left(t\right) {e}^{-{\int }_{{t}_{0}}^{{t}_{1}}\tau \left(s\right)ds}dt,$$

where $c(t)$ is the color at point $t$, $\alpha \left(t\right)$ is the opacity at point $t$, and $\tau \left(s\right)$ is the optical depth or attenuation coefficient at position $s$. The compositing equation used in volume rendering can be expressed as follows [51–53]:

9 $$C= {\sum }_{i}{c}_{i}\cdot {\alpha }_{i} \cdot {\prod }_{j=1}^{i-1}\left(1-{\alpha }_{j}\right)$$

where ${c}_{i}$ is the color of the $i-th$ sample, ${\alpha }_{i}$ is the opacity of the $i-th$ sample, and the product term accounts for the accumulated transparency of all preceding samples.

6) Postrendering

In the postrendering stage, further adjustments and enhancements are made to the rendered output, benefiting significantly from the initial use of the LOD algorithm in the preprocessing phase. This stage focuses on refining the ROIs by rerendering them at the original resolution if downsampling has occurred. Adaptive resolution changes enable selective quality improvement in specific regions [54, 55]. The process involves several steps: storing current camera settings to maintain the user perspective, calculating subvolume dimensions on the basis of downsampled data, cropping the relevant region from the original resolution data, creating a high-resolution subvolume, rerendering the region with its original resolution, and finally restoring camera settings to reflect the updated high-resolution view.

The methodology of the LOD algorithm, which underpins the preprocessing phase and supports these refinements, is provided in Algorithm 1. This pseudocode provides a structured overview of the processes involved, from initialization and volume preparation to progressive LOD rendering and final visualization.

## Validation Methods

To ensure the performance and efficiency of DECODE-3DViz, a comprehensive validation approach comprising analytical evaluation, clinical evaluation, and user feedback via a questionnaire is used. Each method provides a distinct perspective on the system's capabilities, offering a thorough and multifaceted assessment.

### Analytical Evaluation

This evaluation focuses on quantitative performance metrics, including the rendering time (ms), refresh rate (FPS), and GPU memory usage (MB), which are essential for assessing computational efficiency, real-time interactivity, and resource optimization when handling large-scale medical imaging datasets [56, 57]. The rendering time was assessed via browser developer tools (DevTools) available from multiple web browsers. The performance panel within the console was utilized to capture and profile frame execution times, providing a precise evaluation of rendering efficiency. The frame refresh rate (FPS) and GPU memory usage were measured through the rendering tab in DevTools, specifically the Frame Rendering Stats feature.

Algorithm 1.Level of Detail (LOD) Optimization for Efficient WebGL Volume Rendering
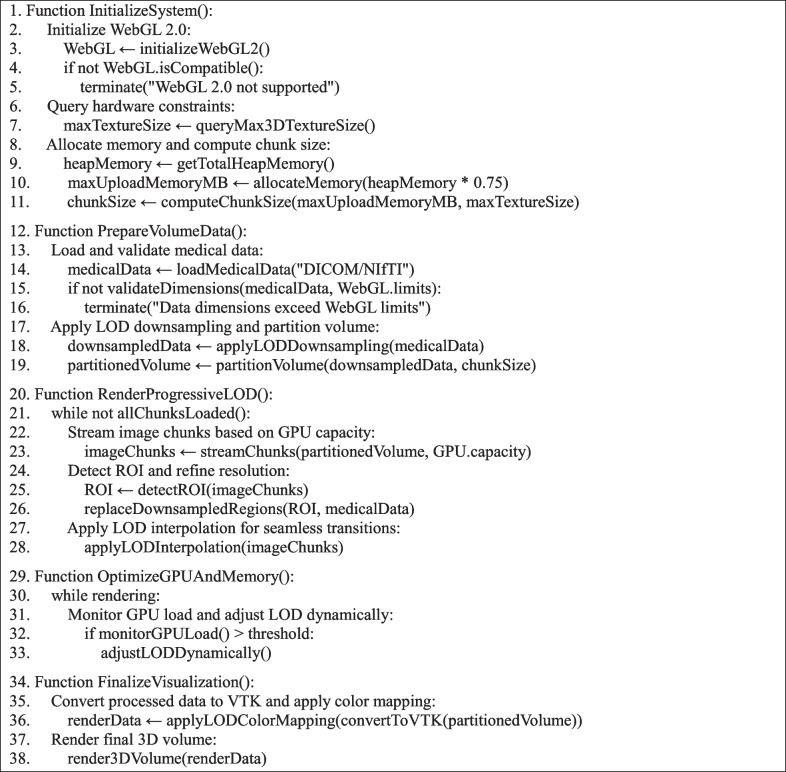


### Clinical Evaluation

This assessment evaluated the system's effectiveness in a real-world clinical setting and involved specialists in vascular and endovascular surgery who utilized DECODE-3DViz to review and diagnose peripheral artery CT images. The clinical evaluation is conducted to determine how well the tool aids in accurate diagnosis, its usability within the clinical workflow, and its overall impact on patient care.

### *User Feedback *via* Questionnaire*

User satisfaction and preferences are gathered through a questionnaire, which compares images generated by DECODE-3DViz with those from other state-of-the-art (SoTA) tools, such as IMAGE-IN [58], BlueLight [6], VolView [14], and Glance [59]. Feedback is collected on several visual characteristics using a Likert scale ranging from 1 (very unsatisfied) to 5 (very satisfied) [60]. The questionnaire covers three main areas: visual characteristics (including structure definition, depth perception, texture appearance, fidelity, and diagnostic ability), reliability ratings, and recommendations. In addition, open-ended questions invite participants to express their preferences for DECODE-3DViz and suggest improvements, providing valuable qualitative feedback to enhance the tool's capabilities. To ensure robust quantitative analysis, the collected data were subjected to statistical evaluation via analysis of variance (ANOVA) [61] to determine the significance of differences in user ratings between the tools. ANOVA was employed to test the null hypothesis that there are no significant differences in the mean ratings across the tools. The *F*-statistic, calculated as the ratio of between-group variance to within-group variance, was used to assess the overall significance of the differences. The between-group variance (SSB) measures the variability of the group means from the overall mean, whereas the within-group variance (SSW) captures the variability of individual ratings within each group. The F-statistic is computed as:

10 $$F=\frac{SSB/\left(k-1\right)}{SSW/\left(N-k\right)}$$

where $k$ is the number of tools, $N$ is the total number of observations, SSB is the sum of squares between groups, and SSW is the sum of squares within groups. The degrees of freedom for the *F*-statistic are ${df}_{1}=k-1$ (between groups) and ${df}_{1}=k-1$ (within groups). The resulting p-value, derived from the *F*-distribution, indicates the probability of observing the data if the null hypothesis is true. A p-value < 0.05 was considered statistically significant, suggesting that at least one tool's mean rating significantly differed from the others.

## Results

To evaluate the performance of DECODE-3DViz, tests were conducted via two systems with distinct hardware specifications. The first system was a laptop running Windows 11 Pro 64-bit, equipped with an Intel(R) Core (TM) i7-11800H CPU at 2.30 GHz with 16 cores, 16 GB of memory, and an NVIDIA GeForce RTX 3070 GPU. The display rate for this system was 144 Hz. The second system was a desktop running Windows 10 Pro 64-bit, featuring an Intel(R) Core (TM) i7-9700F CPU at 3.00 GHz with 8 cores, 32 GB of memory, and an NVIDIA GeForce RTX 3080 GPU. These hardware configurations were selected to assess DECODE-3DViz's performance across both mobile and stationary platforms, providing insights into its ability to handle high-resolution medical imaging data.

### Effects of Visualization Parameters on Peripheral Artery CT Images

DECODE-3DViz uses key visualization parameters, including the sample distance, gradient, and scalar opacity, to significantly enhance the quality of peripheral artery CT images:

- Sample Distance (0.1 to 1): Controls the interval for sampling data points along rays. Lower values provide greater detail and smoother transitions, whereas higher values prioritize performance with reduced detail.
- Gradient (0 to 1): Enhances shading and depth perception, with higher values increasing contrast and highlighting anatomical features such as arteries.
- Scalar Opacity (0 to 255): Regulates transparency, allowing for better visualization of internal structures at lower values and emphasizing specific regions at higher values.

Fine-tuning these parameters enables DECODE-3DViz to deliver high-quality, diagnostic-grade visualizations that enhance image clarity and utility for clinical evaluation.

**Fig. 4:**
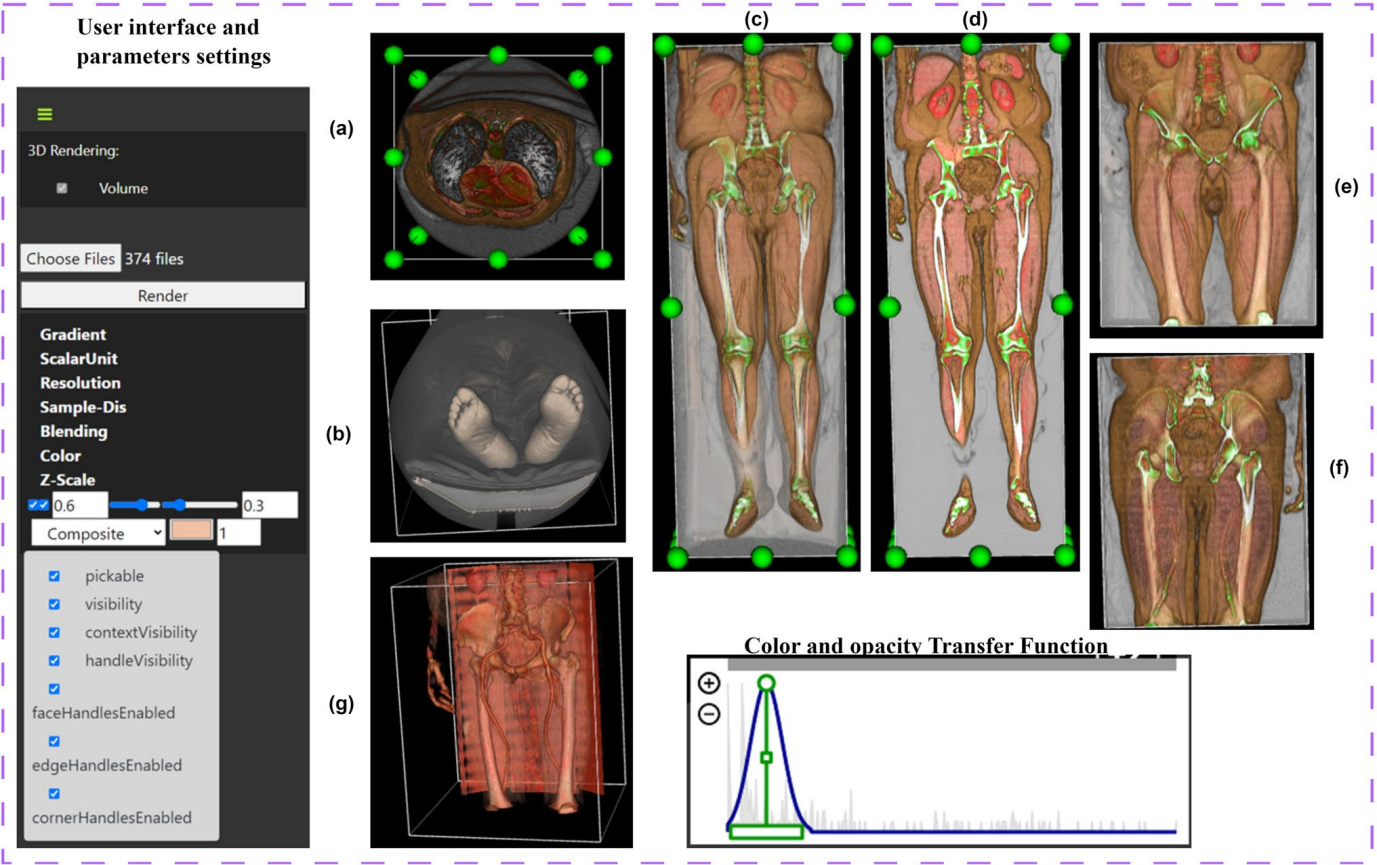
Visualization and Parameter Effects on Peripheral Artery CT Images of Patient #17: (**a**) Top Series Axial View, (**b**) Bottom Series Axial View, (**c**) Coronal View, (**d**) Cropped Coronal View, (**e**) Cropped Front Coronal View of Region of Interest, (**f**) Cropped Back Coronal View of Region of Interest, (**g**) Enhanced Detailed View of Pelvic Arteries with Transfer Function Adjustments

Figure 4 illustrates the effects of various visualization parameters on the CT images of Patient #17, demonstrating the versatility of DECODE-3DViz across different settings:

- Fig. 4(a): High detail rendering with a sample distance of 0.1 and a gradient of 0.6, enhancing peripheral artery visibility with interactive adjustments for regions of interest.
- Fig. 4(b): Focused on the feet, maintaining high detail with the same sample distance and gradient settings as in Fig. 4(a).
- Fig. 4(c): Comprehensive assessment of the peripheral artery system relative to the skeletal structure, using a sample distance of 0.75 and a gradient of 0.6.
- Fig. 4(d): Detailed visualization with strong contrast to differentiate vascular structures, achieved with a sample distance of 0.1 and a gradient of 1.
- Fig. 4(e): Balancing detail and performance, focused on the upper thighs and pelvic region, with a sample distance of 0.5 and a gradient of 1.
- Fig. 4(f) provides detailed, contrasting views of the peripheral arteries using the same parameters as those used in Fig. 4(e).
- Fig. 4(g): Impact of combined parameters (sample distance of 0.3, gradient of 1, and scalar opacity of 170) on visualization quality and detail, particularly for the pelvic arteries.

This comprehensive set of images highlights the versatility and precision of DECODE-3DViz in rendering detailed vascular anatomy.

### Level of Detail Optimization in Large-Scale Data Management

**Fig. 5:**
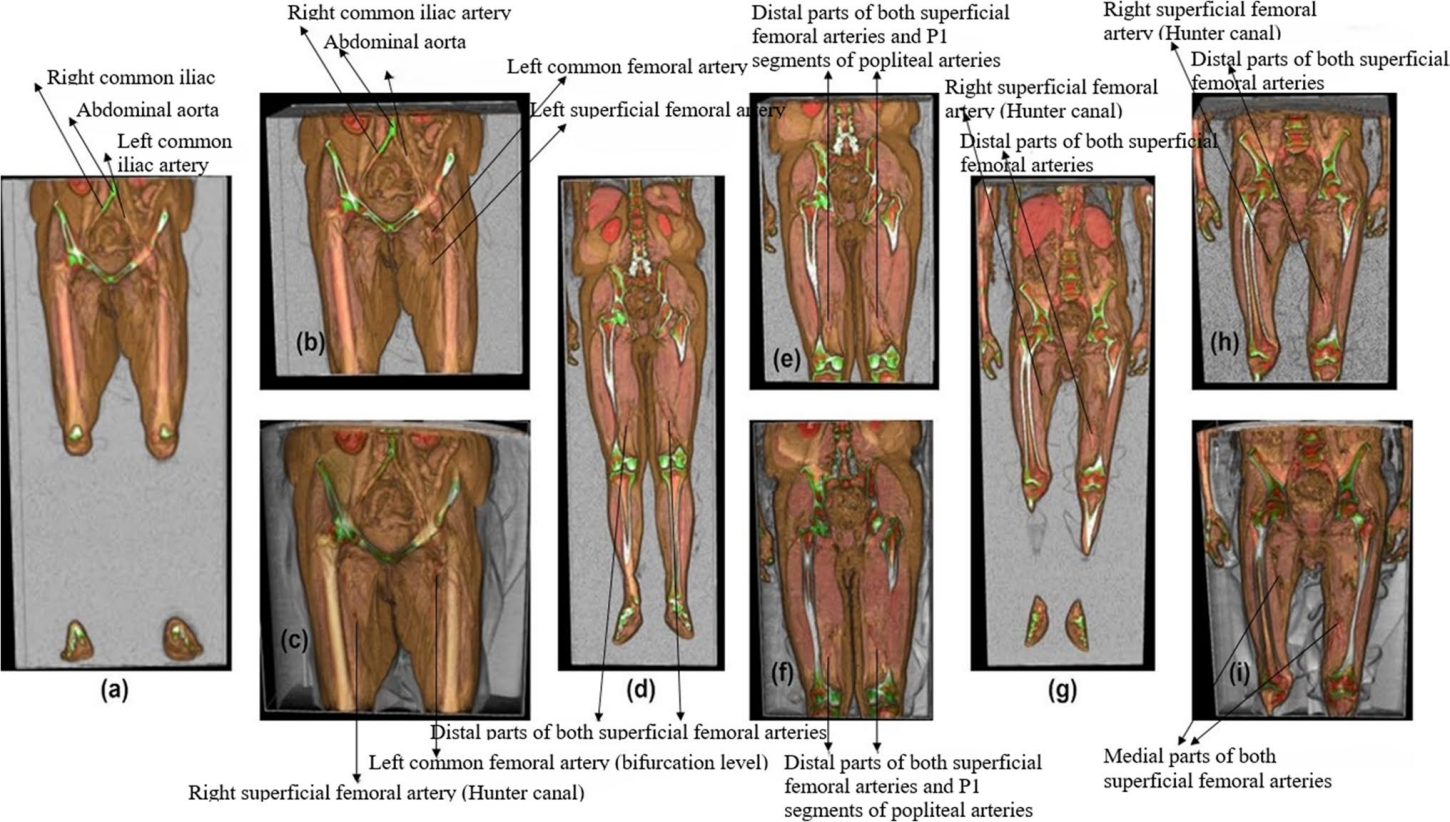
Visualization Results of Progressive Streaming and Level of Detail Volume Rendering for Three Case Studies (a-c: Case Study 1; d-f: Case Study 2; g-i: Case Study 3)

To efficiently manage large volumetric datasets and prevent browser crashes, our methodology partitions the data into manageable chunks, as demonstrated by the use of the laptop and desktop systems in the evaluation and across the three case studies shown in Fig. 5 and Table 1. The application of progressive chunk streaming and LOD-based optimizations has significantly improved the visualization pipeline, directly aligning with the research objectives. The results confirm that DECODE-3DViz successfully overcomes WebGL texture size limitations (RO1), mitigates memory allocation errors (RO2), and implements efficient chunk streaming for large datasets (RO3). In addition, the system dynamically applies downsampling only.

**Table 1:** Parameters and Results of Progressive Streaming and Level of Detail Volume Rendering for Three Case Studies

Parameters	Case study 1	Case study 2	Case study 3
Maximum 3D Texture Size	2048	2048	2048
Computed Chunk Size	512	512	512
Initial JS Heap Size Limit (MB)	4095.75	4095.75	4095.75
Total Chunks to Process	4	5	4
Processed Chunks (1/5)—Used JS Heap Size (MB)	868.8	869.46	871.43
Processed Chunks (2/5)—Used JS Heap Size (MB)	1636.81	1637.48	1639.45
Processed Chunks (3/5)—Used JS Heap Size (MB)	2404.21	2405.53	2407.49
Processed Chunks (4/5)—Used JS Heap Size (MB)	2558.64	3173.04	3164.36
Processed Chunks (5/5)—Used JS Heap Size (MB)	-	3459.37	-
Dimensions of Combined Original Data	[512, 512, 1639]	[512, 512, 2239]	[512, 512, 2041]
Dimensions of Vol_img downsampled	[320, 320, 1024]	[235, 235, 1024]	[257, 257, 1024]
Dimensions of ROI_Vol_img downsampled	[320, 190, 474]	[235, 98, 520]	[257, 124, 492]
Dimensions of ROI_Vol_img original	[512, 304, 761]	[512, 216, 1138]	[512, 248, 982]

when necessary (RO4), preserving high-resolution details for regions of interest (RO5). This structured approach enhances real-time rendering performance while maintaining diagnostic fidelity.

o Case Study 1: This case study, a series for Patient #2, focused on the aortoiliac segment, allowing detailed visualization of the abdominal aorta and iliac arteries. For this case, the dataset was divided on the basis of WebGL's texture size limits and JavaScript heap size, optimizing memory use and maintaining responsiveness. Downsampling with the LOD algorithm was applied only when necessary to preserve crucial details, as it is shown in Fig. 5 (a). The pipeline allows for the rendering of ROIs at their original resolution, enhancing diagnostic capabilities. Figures 5 (b) and (c) illustrate the progression from initial rendering to high-detail rerendering, revealing finer vascular structures crucial for diagnosis

o Case Study 2: This case study, a series involving Patient #17, focused on the femoral arteries, specifically the distal parts of the superficial femoral arteries as they passed through the Hunter canal. As shown in Figs. 5 (d-f), this case study applies similar chunking and downsampling methods to ensure that the intricate anatomical details are clearly visualized. The LOD algorithm enables the detailed rendering of the arteries within their surrounding anatomical context, improving visualization and diagnostic interpretation of arterial segments that are critical for assessing peripheral vascular diseases.
o Case Study 3: This case study, a series for Patient #21, examines the popliteal arterial segment, particularly focusing on the knee area and the P1 segment of the popliteal artery. As it is illustrated in Figs. 5 (g-i), this case study uses chunked and downsampled datasets to maintain high resolution while rendering complex arterial pathways. The rerendered ROIs in this segment provided a precise view of the arterial structures, supporting accurate assessment and planning for interventions.

This progressive streaming and LOD volume rendering approach effectively manages large datasets, minimizes resolution loss, and ensures high-fidelity, interactive 3D visualizations, greatly enhancing the diagnostic accuracy of WebGL-based medical imaging tools.

### Analytical Performance and Evaluation

This section compares the performance of DECODE-3DViz with that of other visualization tools (IMAGE-IN, BlueLight, VolView, and Glance) in the context of 3D WebGL volume rendering for CT peripheral artery images. Key metrics, including render time, FPS, and GPU memory usage, are evaluated across laptop and desktop environments. DECODE-3DViz demonstrates superior efficiency and effectiveness, outperforming the other tools in rendering high-quality medical images.

**Fig. 6:**
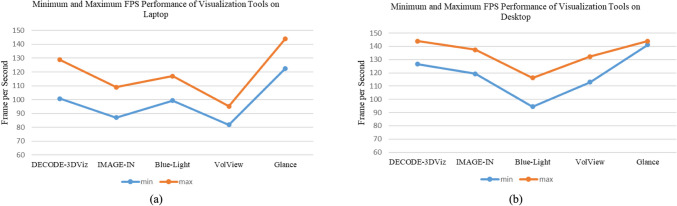
Minimum and Maximum Refresh Rate Performance of Visualization Tools on: (**a**) Laptop and (**b**) Desktop

Table 2 presents a detailed comparison, showing that DECODE-3DViz consistently achieves faster render times, lower GPU memory usage, and robust FPS performance. Figure 6 shows the FPS performance, where DECODE-3DViz maintains a high FPS on both laptops (Fig. 6(a)) and desktops (Fig. 6(b)), highlighting its ability to deliver smooth and fluid visualizations. In particular, DECODE-3DViz outperforms IMAGE-IN and BlueLight, whereas Glance has the highest FPS, indicating superior optimization on desktops.

**Table 2:** Analytical Performance Evaluation Metrics of DECODE-3DViz and State-of-the-Art Visualization Tools for 3D WebGL Volume Rendering (mean ± std)

Tool	DECODE-3DViz	IMAGE-IN	BlueLight	VolView	Glance
Laptop Render Time (ms)	26.88 ± 2.65	41.35 ± 3.88	604.06 ± 108.73	42.73 ± 4.74	73.74 ± 5.61
Desktop Render Time (ms)	48.07 ± 2.36	79.57 ± 3.76	688.04 ± 39.35	81.52 ± 5.32	106.93 ± 4.27
Laptop Refresh Rate (FPS)	115.2	108.5	113.4	90.4	139.5
Desktop Refresh Rate (FPS)	134.03	128.95	110.89	120.99	143.57
Laptop GPU memory usage (MB)	3.4	7	108.9	14.4	20
Desktop GPU memory usage (MB)	2.5	4.12	108.6	11.03	22.61

### Clinical Evaluation

The DECODE-3DViz tool uses an LOD algorithm, including the processes of rendering and rerendering regions of interest (ROIs), to provide detailed and clinical views of peripheral arterial segments. This capability is crucial for evaluating potential operative strategies and enhancing diagnostic accuracy. In Case Study 1 (Fig. 5(a-c)), DECODE-3DViz facilitated clear visualization of the aortoiliac segment, effectively differentiating adjacent structures. This visualization provided critical insights into the diameters and wall morphology of the arteries and the extent of atherosclerotic, primarily calcified, lesions. In addition, the tool offered a clear view of both larger and smaller arterial branches, particularly around the femoral bifurcation. This is advantageous for planning surgical dissections and assessing the development of the collateral network.

In Studies 2 (Figs. 5(d-f)) and 3 (Figs. 5(g-i)), the focus was on the femoral‒popliteal arterial segment, specifically the distal part of the superficial femoral artery, as it traverses the adductor (Hunter) canal and the P1 segment of the popliteal artery. DECODE-3DViz demonstrated its ability to track the arterial pathway and its anatomical relationships with high fidelity and texture quality. This detailed level of visualization is instrumental in assessing and planning strategies for treating occlusive disease in the femoral‒popliteal segment. The rerendered ROIs, processed through the LOD algorithm, provided a more precise view of the arterial structures and their relationships with surrounding tissues, enhancing the tool's clinical utility in medicine.

The ability of DECODE-3DViz to adapt to other vascular structures supports its potential use in coronary artery imaging. The system's selective rerendering ensures high-resolution visualization of regions requiring detailed assessment, making it suitable for evaluating aneurysms, stenotic lesions, and vessel integrity in different anatomical contexts. In addition, its ability to process large volumetric datasets with adaptive resolution allows its extension to other anatomical regions, such as thoracic aortic dissection assessment. Furthermore, the system’s ability to distinguish between high-density structures makes it well suited for bone visualization, including orthopedic assessments, fracture detection, and skeletal deformity analysis. This adaptability highlights its potential in neurology, cardiology, orthopedics, and oncology for precise 3D visualization of complex anatomical structures.

### Questionnaire and Assessment Protocol

The evaluation of DECODE-3DViz was conducted with a cohort of 12 participants (four from the University of Ioannina, Greece; one from the University of Patras, Greece; one from the University of Milan, Italy; two from the University of Kragujevac, Serbia; one from the University of Montpellier, France; and three from AGH University of Krakow, Poland) from various professional backgrounds, including researchers, software engineers, PhD students, biomedical engineers, professors, and clinicians. The participants reviewed and assessed images generated by DECODE-3DViz alongside other state-of-the-art tools (IMAGE-IN, BlueLight, VolView, and Glance), providing comprehensive feedback on multiple visual attributes, as detailed in Table 3.

1. Visual characteristics

**Table 3:** Likert’s scale evaluation of volume rendering across Peripheral arteries structures and characteristics from the DECODE-3DViz and State-of-the-Art Tools

Characteristics	Tools	Iliac Artery	Femoral Artery	Popliteal Artery	Tibial Artery	Mean ± SD
Definition of Structure	DECODE-3DViz	4.5	4.41	4.41	4.16	4.37 ± 0.15
	IMAGE-IN	2.91	2.6	2.68	2.53	2.68 ± 0.16
	BlueLight	2.2	2.2	2.05	2.05	2.12 ± 0.08
	VolView	3.17	3.42	3.31	3.36	3.31 ± 0.11
	Glance	3.59	3.75	3.6	3.97	3.72 ± 0.16
Depth Perception	DECODE-3DViz	4.33	4.41	4.16	4.16	4.26 ± 0.12
	IMAGE-IN	2.85	2.55	2.6	2.3	2.57 ± 0.14
	BlueLight	2.15	2.2	2.11	2.04	2.12 ± 0.07
	VolView	3.2	3.45	3.35	3.35	3.31 ± 0.11
	Glance	3.6	3.8	3.7	4.0	3.77 ± 0.16
Texture Appearance	DECODE-3DViz	4.25	4.25	4	4	4.12 ± 0.12
	IMAGE-IN	2.91	2.6	2.68	2.53	2.68 ± 0.16
	BlueLight	2.15	2.1	2.07	2.1	2.10 ± 0.07
	VolView	3.2	3.45	3.35	3.35	3.31 ± 0.11
	Glance	3.6	3.8	3.7	4.0	3.77 ± 0.16
Fidelity	DECODE-3DViz	4.41	4.41	4.25	4.16	4.30 ± 0.12
	IMAGE-IN	2.91	2.6	2.68	2.53	2.68 ± 0.16
	BlueLight	2.10	2.0	2.11	2.04	2.08 ± 0.07
	VolView	3.17	3.42	3.31	3.36	3.31 ± 0.11
	Glance	3.59	3.75	3.6	3.97	3.72 ± 0.16
Diagnostic Ability	DECODE-3DViz	4.0	4.0	3.75	3.75	3.87 ± 0.12
	IMAGE-IN	2.91	2.6	2.68	2.53	2.68 ± 0.16
	BlueLight	2.0	2.2	2.14	1.94	2.02 ± 0.10
	VolView	3.5	3.4	3.3	3.3	3.45 ± 0.11
	Glance	3.6	3.8	3.6	3.9	3.67 ± 0.16

DECODE-3DViz consistently outperformed other tools across all visual characteristics. Statistical analysis using ANOVA revealed significant differences in user ratings between the tools. For the definition of structure, the ANOVA results $F\left(\text{4,15}\right)=164.44, p<0.001,$ indicated that DECODE-3DViz (mean = 4.37 ± 0.15) significantly outperformed IMAGE-IN (mean = 2.68 ± 0.16), BlueLight (mean = 2.12 ± 0.08), VolView (mean = 3.31 ± 0.11), and Glance (mean = 3.72 ± 0.16). Similarly, for depth perception, DECODE-3DViz excelled (mean = 4.26 ± 0.12) in representing spatial relationships within volumetric data, enhancing the understanding of complex anatomical features. The participants also praised DECODE-3DViz for its texture appearance (mean = 4.12 ± 0.12), noting the realistic surface textures that improved the visual realism and quality of medical images. In terms of fidelity, DECODE-3DViz demonstrated a high level of accuracy (mean = 4.30 ± 0.12) in depicting real peripheral artery tissue, which is crucial for diagnostic reliability. Finally, for diagnostic ability, DECODE-3DViz (mean = 3.87 ± 0.12) provided more diagnostically useful visualizations than the other tools did, further enhancing its utility in medical applications. The ANOVA results confirmed these differences as statistically significant ($p<0.001$ for all characteristics), underscoring the superior performance of DECODE-3DViz.

2) Additional Questions

Reliability of DECODE-3DViz: Participants rated the reliability at 4.41, which was significantly higher than the SoTA average of 2.66 $\left(p<0.001\right)$, indicating strong confidence in its performance and consistency. Recommendation of DECODE-3DViz: DECODE-3DViz received a high recommendation score of 4.5, whereas the SoTA average was 2.66 $\left(p<0.001\right)$, reflecting strong user preference and high satisfaction.

3) Open-Ended Questions

The participants highlighted several key strengths, such as the tool's reliability and superior performance on large datasets, producing clear and high-quality visualizations of the peripheral vasculature. They noted the detailed and accurate representation of anatomy and pathology, particularly in viewing the iliac and femoral arteries. Suggestions for improvement included adding rendering filters for visualizing different tissue types, which would increase the utility of DECODE-3DViz in medicine.

## Discussion

### Principal Results

**Fig. 7:**
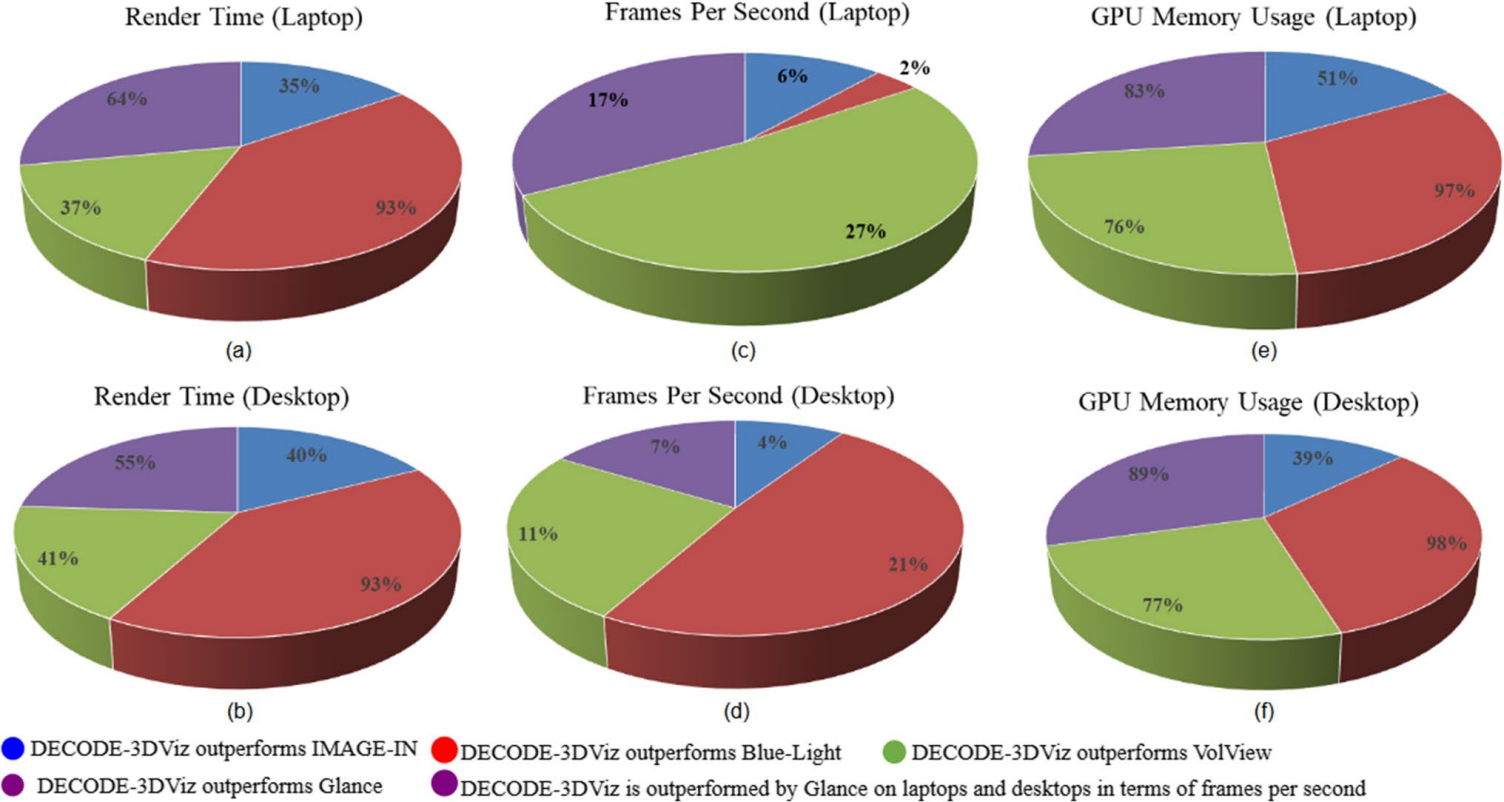
Performance Comparison of DECODE-3DViz and State-of-the-Art Visualization Tools which are IMAGE-IN, BlueLight, VolView, and Glance. (**a**) Render Time on Laptop, (**b**) Render Time on Desktop, (**c**) Frames Per Second (FPS) on Laptop, (**d**) FPS on Desktop, (**e**) GPU Memory Usage on Laptop, and (**f**) GPU Memory Usage on Desktop

The performance of DECODE-3DViz was evaluated against SoTA visualization tools, including IMAGE-IN, BlueLight, VolView, and Glance, which focus on key metrics such as the rendering time, refresh rate (FPS), and GPU memory usage across both laptop and desktop environments. As shown in Table 2 and Fig. 7, the evaluation was conducted on low- and medium-sized series of the dataset, ensuring a thorough performance assessment before applying the method to larger-scale series of the dataset. DECODE-3DViz demonstrated a 93% improvement in rendering time over BlueLight on both laptops (Fig. 7(a)) and desktops (Fig. 7(b)) while also outperforming IMAGE-IN, VolView, and Glance. In terms of the refresh rate, DECODE-3DViz maintains a high FPS across devices, surpassing IMAGE-IN and VolView while performing competitively with glance (Fig. 7(c-d)). This consistency ensures seamless interaction for real-time visualization, which is essential for clinical applications and diagnostic precision. In addition, DECODE-3DViz achieves superior GPU memory efficiency, significantly reducing memory usage compared with BlueLight and maintaining substantial reductions in desktops (Fig. 7(e–f)). These optimizations position DECODE-3DViz as a highly efficient and scalable solution for web-based visualization of peripheral artery CT images, ensuring real-time rendering even on resource-constrained hardware.

### Comparative Evaluation of DECODE-3DViz and Other Modalities on Large-Scale Dataset

The evaluation of DECODE-3DViz against other SoTA visualization tools, using large-scale CT datasets from patients #5 (series 6) and #7 (series 5), is shown in Table 4 and detailed in Table 5.

1. Rendering Efficiency and Performance

**Table 4:** Large-Size Dataset Specification for DECODE-3DViz validation and comparison with others

Patient ID	Dimension	Size	Bits stored	Slice thickness	Spacing bet slice	Pixel spacing (mm)
5	(512, 512, 2299)	1.13 GB	16	0.625 mm	0.625 mm	0.955078
7	(512, 512, 2305)					0.912109

**Table 5:** Evaluation Metrics of DECODE-3DViz and State-of-the-Art Visualization Tools on Large-Size Dataset (mean ± std)

Laptop	Metrics	Patient ID	DECODE-3DViz	IMAGE-IN	BlueLight	VolView	Glance
	Render Time (ms)	5	41.8 ± 4.49	x	x	x	x
		7	38.4 ± 1.20	x	2559 ± 157.02	x	x
	Refresh Rate (FPS)	5	138.74 ± 4.94	x	x	x	x
		7	142.0 ± 1.47	x	96.38 ± 24.62	x	x
	GPU memory usage (MB)	5	3.14	x	x	x	x
		7	3.1	x	94.7	x	x
Desktop	Render Time (ms)	5	54.6 ± 2.65	x	x	x	x
		7	53.8 ± 2.85	x	3185.4 ± 394.7	x	x
	Refresh Rate (FPS)	5	135.62 ± 3.09	x	x	x	x
		7	129.06 ± 8.92	x	58.6 ± 19.96	x	x
	GPU memory usage (MB)	5	2.6	x	x	x	x
		7	2.6	x	101.64	x	x

DECODE-3DViz significantly outperforms other tools, especially BlueLight, by reducing rendering times by approximately 98% on both laptops and desktops. It also provided smoother and more responsive visualizations, with FPS improvements of 44% on laptops and 131% on desktops.

2) GPU memory usage and resource efficiency

Compared with BlueLight, efficiency, with 96.7% less memory on laptops and 97.4% less memory on desktops. This efficiency enhances the performance on less powerful hardware, expanding the accessibility of DECODE-3DViz.

3) Challenges in Processing Large-Scale Datasets

VolView experienced a "range error: invalid array buffer length," indicating issues with large datasets. IMAGE-IN and Glance failed to render these datasets, whereas BlueLight successfully rendered Patient #7's dataset. These difficulties, indicated by the 'x' values in Table 5, underscore the limitations of these tools due to high computational demands and inadequate memory management, in contrast with DECODE-3DViz's robust handling of extensive data volumes.

### Innovations and Robustness in DECODE-3DViz

DECODE-3DViz introduces significant advancements in web-based medical imaging visualization, particularly for handling large-scale datasets such as peripheral artery CT images. The system overcomes WebGL texture size limitations and browser memory constraints, which have traditionally impeded real-time visualization. Key innovations include progressive chunk streaming and dynamic LOD algorithms, optimizing memory usage and enabling high-resolution rendering tailored to user interactions and the importance of specific regions. These features ensure smooth, detailed visualizations crucial for accurate diagnostics. DECODE-3DViz outperforms other SoTA methods in rendering time, refresh rate, and GPU memory usage, highlighting superior GPU resource management. Its robust performance has been validated through comprehensive evaluations, confirming its efficiency in complex medical imaging tasks. Moreover, DECODE-3DViz's web-based accessibility distinguishes it from traditional tools, broadening the availability of high-quality medical visualizations to medical professionals and patients.

### Clinical Impact of DECODE-3DViz in Real-Word Clinical Setting

DECODE-3DViz enhances diagnostic precision and intervention planning by providing high-resolution, interactive views of peripheral artery CT images. Clinicians can assess complex pathologies such as atherosclerosis and stenosis with clarity, improving diagnostic accuracy and treatment efficacy. Surgical planning enables precise evaluation of arterial segments, facilitating accurate measurement of diameters and wall morphology, which is critical for assessing lesion severity and optimizing procedures. The tool supports noninvasive monitoring of disease progression and postoperative recovery by depicting arterial pathways with high fidelity. Its integration into digital workflows enhances real-time collaboration and multidisciplinary case discussions, streamlining decision-making in vascular diagnostics. By optimizing the diagnostic workflow, DECODE-3DViz accelerates image interpretation, reducing the time required for radiologists and vascular specialists to reach conclusions. This efficiency benefits triage and urgent interventions, ensuring quicker treatment decisions. Seamless integration with picture archiving and communication systems (PACSs) minimizes workflow disruptions, whereas enhanced visualization capabilities support telemedicine, allowing remote specialists to review cases efficiently and expand access to expert-driven diagnosis and treatment planning. Furthermore, DECODE-3DViz enables visualization of both vascular and osseous structures, enhancing orthopedic and vascular assessments by allowing simultaneous evaluation of arterial integrity and skeletal conditions. The advanced rendering pipeline ensures precise differentiation of vascular calcifications, fractures, and soft tissue structures, making it an effective tool for preoperative planning and post-treatment evaluation.

DECODE-3DViz significantly enhances the detection and assessment of vascular abnormalities, including arterial stenosis, occlusions, and calcifications, by providing clinicians with detailed, high-resolution images for accurate interpretation. The ability to visualize both large arterial structures and finer vascular branches ensures a comprehensive evaluation of PAD. One of DECODE-3DViz’s most impactful clinical applications is its role in telemedicine and remote diagnostics. The system's accessibility allows specialists to review cases remotely and provide expert opinions from various geographic locations. This feature is particularly beneficial for hospitals with limited vascular imaging expertise, enabling remote consultations with specialists to improve patient outcomes.

### Limitations and Future Work

DECODE-3DViz currently processes datasets only in DICOM and NIfTI formats. In addition, advanced rendering filters for tissue differentiation, which are vital for distinguishing between similar intensity tissues such as bone, muscle, arterial, and adipose tissue, are lacking. Expanding the format support and integrating rendering filters would enhance the applicability and versatility of DECODE-3DViz, providing a more comprehensive solution for medical imaging visualization and analysis. Future work will focus on enhancing real-time volumetric rendering by integrating adaptive transfer functions to improve tissue segmentation and classification. Moreover, incorporating AI-driven automation for feature extraction and anomaly detection could further streamline clinical decision-making. A key focus will be on automated peripheral artery risk classification, leveraging machine learning models to assess arterial stenosis severity and predict potential occlusions. This automated risk assessment could aid clinicians in early disease detection and personalized treatment planning, reducing the likelihood of adverse cardiovascular events. Expanding cloud-based capabilities will facilitate multiuser collaboration and enable seamless access to imaging data across institutions, supporting broader clinical adoption and interoperability within modern healthcare infrastructures.

## Conclusions

The development and implementation of DECODE-3DViz have enabled significant progress in medical imaging, particularly in visualizing peripheral artery CT images. This system adeptly addresses key challenges in rendering large-scale medical datasets on web platforms, overcoming WebGL texture size constraints and browser memory limitations. By using advanced techniques such as data chunk streaming and the level of detail (LOD) algorithm, DECODE-3DViz dynamically adjusts resolution on the basis of user interaction and the importance of visualized regions, increasing both user engagement and visualization quality. This capability is invaluable for clinical experts, as it enhances diagnostic accuracy and supports detailed preoperative planning by delivering high-fidelity visualizations of complex vascular structures. The ability to manage extensive datasets without sacrificing performance or visual fidelity sets DECODE-3DViz apart from existing solutions, offering a more robust and user-friendly tool for medical professionals. Future developments include the integration of advanced rendering filters for visualizing different tissue types, such as bone, muscle, and adipose tissue, thereby broadening the system's applicability across various medical scenarios. In addition, further optimization is needed to improve the system's scalability and efficiency in handling even larger datasets.

## Supplementary Information

Below is the link to the electronic supplementary material.

Supplementary file1 (XLSX 35 KB)

Supplementary file2 (XLSX 40 KB)

## Data Availability

The dataset analyzed during this study are available from the corresponding author on reasonable request.
